# A Novel Signal Separation Method Based on Improved Sparse Non-Negative Matrix Factorization

**DOI:** 10.3390/e21050445

**Published:** 2019-04-28

**Authors:** Huaqing Wang, Mengyang Wang, Junlin Li, Liuyang Song, Yansong Hao

**Affiliations:** 1 College of Mechanical & Electrical Engineering, Beijing University of Chemical Technology, Chao Yang District, Beijing 100029, China; 2 Beijing Key Laboratory of High-end Mechanical Equipment Health Monitoring and Self-Recovery, Beijing University of Chemical Technology, Beijing 100029, China

**Keywords:** Sparse non-negative matrix factorization, underdetermined blind source separation, compound faults diagnosis, time–frequency distribution

## Abstract

In order to separate and extract compound fault features of a vibration signal from a single channel, a novel signal separation method is proposed based on improved sparse non-negative matrix factorization (SNMF). In view of the traditional SNMF failure to perform well in the underdetermined blind source separation, a constraint reference vector is introduced in the SNMF algorithm, which can be generated by the pulse method. The square wave sequences are constructed as the constraint reference vector. The output separated signal is constrained by the vector, and the vector will update according to the feedback of the separated signal. The redundancy of the mixture signal will be reduced during the constantly updating of the vector. The time–frequency distribution is firstly applied to capture the local fault features of the vibration signal. Then the high dimension feature matrix of time–frequency distribution is factorized to select local fault features with the improved SNMF method. Meanwhile, the compound fault features can be separated and extracted automatically by using the sparse property of the improved SNMF method. Finally, envelope analysis is used to identify the feature of the output separated signal and realize compound faults diagnosis. The simulation and test results show that the proposed method can effectively solve the separation of compound faults for rotating machinery, which can reduce the dimension and improve the efficiency of algorithm. It is also confirmed that the feature extraction and separation capability of proposed method is superior to the traditional SNMF algorithm.

## 1. Introduction

The analysis methods based on a vibration signal have been diffusely used in the fault diagnosis of mechanical equipment [1,2], because the vibration signal usually contains the main information of the operating state about the equipment [3,4]. However, the observed signals of mechanical equipment are often non-stationary [5,6], and accompanied with multiple fault characteristics at the same time in real engineering [7,8]. Moreover, the coupling of fault features also increases the difficulty of compound faults diagnosis [9,10]. Therefore, it is of great significance for the normal operation of mechanical equipment and healthy system management to separate out multiple source signals and extract compound fault features effectively from vibration signals [11,12].

A transform domain decomposition method [13] is usually used to achieve the separation of multiple source components, such as empirical mode decomposition (EMD) [14], local mean decomposition (LMD), and variational mode decomposition (VMD). Non-negative matrix factorization (NMF), as a new feature extraction algorithm, gives physical meaning regarding the decomposition form and result, which overcomes the defects of traditional algorithms, has been diffusely used in the fields of digital images processing [15], biomedical engineering [16], machine learning [17,18], computer vision [19], information retrieval [20], hyperspectral unmixing [21,22], and etc.

With the deepness of research, the NMF algorithm is focused on the blind source separation (BSS) problem [23]. Compared with independent component analysis (ICA) and sparse component analysis (SCA), the NMF algorithm requires fewer constraints, faster convergence, and higher decomposition efficiency [24,25]. However, when the number of observed signals collected by the sensor is less than the number of fault sources, that is, the underdetermined blind source separation problem [26], the NMF algorithm cannot be performed directly, and the improper selection of the loss function about the NMF algorithm also increases the difficulty of signal separation [27]. Hence, how to choose the loss function of the NMF algorithm to realize the underdetermined blind source separation is a major problem to be solved urgently. During the past decade, there has been an increasing interest in the relationship between compound faults diagnosis of mechanical equipment and the development of certain BSS [28], including ICA, SCA, and NMF. Many investigators proposed approaches based on EMD, ensemble empirical mode decomposition (EEMD), VMD, and other algorithms to expand the source signal into multiple virtual channels and selected appropriate components according to relevant information for the underdetermined blind source separation problem [29,30]. For example, Hao et al. [31] introduced a method combining with the intrinsic characteristic-scale decomposition (ICD) and SCA algorithm to realize compound faults diagnosis with single channel signal. Jiang et al. [32] also introduced a method of the EMD-ICA algorithm to separate the end member spectra with a single mixed pixel spectrum. Wang et al. [33] proposed a novel approach of EEMD-ICA algorithm to separate the compound faults. Tang et al. [34] developed a technique based on VMD-ICA algorithm to achieve the underdetermined blind source separation of bearings. In addition, some scholars are committed to the study of NMF algorithm for blind source separation problem. Mirzaei et al. [35] put forward a model on the basis of the Bayesian NMF that achieves the BSS problem of reverberant audio signals. Abdali et al. [36] used the regularized NMF method to realize the BSS problem of music signals with a single channel. Canadas et al. [37] adopted a method of frequency domain clustering non-negative matrix factorization to bring about the separation and extraction of cardiopulmonary signals. Meanwhile, with the popularity of artificial intelligence, the combination of deep learning [38] and the NMF algorithm is also becoming a research hotspot. For example, Nie et al. [39] combined the NMF algorithm and deep learning to achieve speech separation.

The theory of the NMF algorithm has achieved a lot of remarkable results from the proposed, covering a wide range of applications, but still has some room for improvement. In addition, the method based on non-negative matrix factorization is a new field to solve the underdetermined blind source separation problem in the compound faults diagnosis of rotating machinery. The objective function of the traditional NMF algorithm has a single constraint condition, and the signal generated by the actual fault is poorly applied. The data is often redundant after factorization by the algorithm, and the coupling fault features cannot be effectively separated.

Therefore, in this paper, the compound fault features of rolling bearing can be separated and extracted by using the advantage of the improved sparse non-negative matrix factorization. The time–frequency distribution is firstly applied to describe the instant component of vibration signals by combining the time with frequency information in a two-dimensional representation [40]. Typical time–frequency distribution methods include the wavelet transform (WT), the short-time Fourier transform (STFT), the Wigner-Ville distribution and the Hilbert-Huang transform, etc. The short-time Fourier transform is chosen because of its local capacity and simple principle, which makes fault diagnosis much easier and more reliable [41]. According to the feature information of the signal, the constraint reference vectors are constructed by the pulse method. The square wave sequences with the same length as the test signal are generated as the constraint reference vectors. By introducing appropriate reference constraints on the basis of the sparse non-negative matrix factorization (SNMF) algorithm, the improved SNMF method can improve the processing ability of the SNMF algorithm and reduce the procedure of source signals and noise features detected. With the improved SNMF method, the high dimension feature matrix of time–frequency distribution is factorized to select local fault features. Furthermore, according to the sparse property of improved SNMF, the proposed method can accomplish the separation of the compound fault features automatically.

The remaining sections are organized as follows: Section 2 describes the fundamental principle of NMF algorithm. In Section 3, the improved SNMF algorithm is introduced. The separation of compound fault signals based on the proposed method is presented in Section 4. The simulation and test signals are discussed to evaluate the proposed method in Section 5. Finally, the conclusions are summarized in Section 6.

## 2. Principle of Non-Negative Matrix Factorization

Matrix factorization is an effective means for large-scale data processing and analysis. The NMF algorithm can be represented as follows: Given a non-negative matrix $V\in R_{+}^{m\times n}$, the algorithm constructs approximate factorizations of the form with a product of two non-negative matrices $W\in R_{+}^{m\times r}$ and $H\in R_{+}^{r\times n}$ [42], namely:

(1) $$V_{m\times n}\approx W_{m\times r}H_{r\times n}$$

where m is the dimension of the matrix $V_{m\times n}$, and *n* is the number of samples. The parameter r is generally chosen as r<mn/(m+n) and is called reduced rank, thus the product $W_{m\times r}H_{r\times n}$ can be considered as a compressed form of the data $V_{m\times n}$. Varieties of optimization algorithms about loss function were proposed for improving the efficiency of the algorithm, since the NMF algorithm has been put forward. Traditionally, the loss function is represented:

(2) $$\begin{array}{l} D(V||WH)={\left\|V-WH\right\|}^{2}\\ s.t.W,H>0 \end{array}.$$

The NMF algorithm for the loss function of Equation (2) can be regarded as the following optimization problem:

(3) $$\min\;{\left\|V-WH\right\|}_{F}^{2}=\sum_{ij}[v_{ij}-{\left(WH\right)}_{ij}{]}^{2}.$$

The optimization problem in Equation (3) is convex with respect to W and H respectively. However, it is non-convex about the matrices W and H simultaneously. Therefore, the above problem can deal with an iterative multiplicative updated algorithm until the objective function converges to some constant value. The updated rules are presented:

(4) $$w_{ik}\leftarrow w_{ik}\frac{{\left(VH^{T}\right)}_{ik}}{{\left(WHH^{T}\right)}_{ik}},\;h_{ik}\leftarrow h_{ik}\sum_{i}\frac{{\left(W^{T}V\right)}_{kj}}{{\left(W^{T}WH\right)}_{kj}}.$$

Later, the objective function is defined based on the KL divergence, namely:

(5) $$\begin{array}{l} \min\;D(V||WH)=\sum_{ij}[v_{ij}\log\frac{v_{ij}}{{\left(WH\right)}_{ij}}-v_{ij}+(WH{)}_{ij}]\\ s.t.W,H>0 \end{array},$$

So, the update rules are given to obtain ***W*** and ***H***:

(6) $$w_{ik}\leftarrow w_{ik}\frac{\sum_{j}\frac{h_{kj}v_{ij}}{{\left(WH\right)}_{ij}}}{\sum_{u}h_{ku}},\;h_{kj}\leftarrow h_{kj}\frac{\sum_{i}\frac{w_{ik}v_{ij}}{{\left(WH\right)}_{ij}}}{\sum_{v}w_{vk}}.$$

## 3. Basic Principle

### 3.1. Sparse Non-Negative Matrix Factorization

In this section, the basic description of sparseness is discussed.

The idea of “sparse coding” is a sparse representation scheme that can effectively represent typical data vectors with only a few units. In other word, the sparse representation scheme actually means that most of the representation units are close to zero, and only a very small part takes significantly non-zero values.

Currently, there are many functions for measuring the sparseness of data. A normalized scale should have such a feature: The sparsest vector with only a component being non-zero and other components being zero should have a sparseness of one; the least sparsest vector with all elements equal should have a sparseness of zero.

The sparsity degree function [43] in this paper is based on the $L_{1}$ norm namely:

(7) $$sparseness(x)=\frac{\sqrt{n}-(\sum \left|x_{i}\right|)/\sqrt{\sum x_{i}^{2}}}{\sqrt{n}-1},$$

where n is the dimension of the vector x. This function takes a value of 1 if and only if the vector x contains a non-zero element, and takes a value of 0 if and only if all elements are equal, otherwise the values can be smoothly distributed between the two extremes.

**Figure 1:**
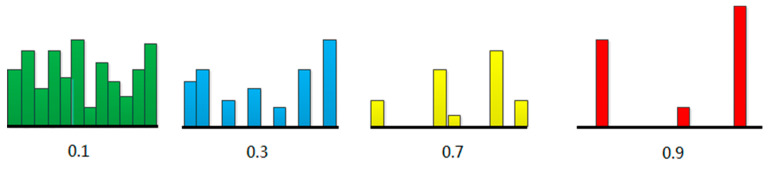
Illustration of various degrees of sparseness.

The illustration of different degrees of sparseness are shown in Figure 1, displaying the sparseness of different levels. Each bar indicates the value of one element. Where the leftmost is at low levels of sparseness, all the elements are substantially equal. The rightmost is at high levels, and most coefficients are zero except for a few non-zero elements.

For the choice of constraint terms, whether it is the sparsity of the constraint ***W***, or the sparsity of the constraint ***H***, or the sparsity of both constraints depends on the specific application in question. Since the base matrix ***W*** contains the feature information of the original data, the sparseness constraint on ***W*** may improve the convenience of storage and calculation, but it may cause the base feature to be missing. Therefore, the coefficient matrix ***H*** is usually constrained effectively, which can effectively enhance the feature of the base matrix W.

The SNMF method based on the *L*_1_ norm constraint is derived from Hoyer’s non-negative sparse coding method [44], which combines the Euclidean distance with the norm constraint to form the objective function.

(8) $$\begin{array}{l} \min\;D(V||WH)={\left\|V-WH\right\|}_{F}^{2}+\lambda {\left\|H\right\|}_{1}\\ s.t.\;W,H>0,\;\left\|a_{i}\right\|=1 \end{array},$$

where λ is a regularization parameter, which is used to balance sparseness and reconstruction error. $a_{i}$ is the *i*th line of ***V***.

The updated rules are determined by:

(9) $$\begin{array}{l} W_{ik}\leftarrow W_{ik}/\sqrt{\sum_{ik}W_{ik}{}^{2}}\;\;\;\;H_{kj}\leftarrow \frac{H_{kj}{\left(W^{T}V\right)}_{kj}}{{\left(W^{T}WH\right)}_{kj}+\lambda }\\ W_{ik}\leftarrow W_{ik}-\eta ((WH-V)H^{T}{)}_{ik} \end{array},$$

where η is the step size of iteration.

Similarly, we can also obtain the objective function, combining the generalized KL (Kullback–Leibler) divergence with the norm constraint,

(10) $$\begin{array}{l} \min\;D(V||WH)=\sum_{ij}[v_{ij}\log\frac{v_{ij}}{{\left(WH\right)}_{ij}}-v_{ij}+(WH{)}_{ij}]+\lambda \sum_{kj}H_{kj}\\ s.t.\;W,H>0,\sum_{i}W_{ik}=1 \end{array}.$$

The updated rules are determined by:

(11) $$\begin{array}{l} W_{ik}\leftarrow \frac{W_{ik}\sum_{j}V_{ij}\frac{H_{kj}}{\sum_{l}W_{il}H_{lj}}}{\sum_{j}H_{kj}}\\ W_{ik}\leftarrow W_{ik}/\sum_{i}W_{ik} \end{array}$$

(12) $$H_{kj}\leftarrow \frac{H_{kj}\sum_{i}V_{ij}\frac{W_{ik}}{\sum_{l}W_{il}H_{lj}}}{1+\lambda }.$$

### 3.2. Improved Sparse Non-Negative Matrix Factorization

In this section, it is shown how to improve sparseness in the SNMF framework.

By comparing the Euclidean distance and KL divergence functions respectively, the *L*_1_ norm is added as the objective function of the sparse constraint term. The convergence of two different objective functions and the certainty of the solution are also analyzed and proved strictly by scholars [45], which provides a solid mathematical theoretical basis for its solution process. However, the solution process based on KL divergence is a multiplicative rule completely, which can reduce the computational complexity and guarantee the process of iteration better [46].

Therefore, the objective function based on KL divergence is chosen as the algorithm. Meanwhile, in order to improve the processing ability of the sparse non-negative matrix factorization algorithm, lower the dimension of the problem, and reduce the redundancy of the information after decomposition, we introduce a constraint reference vector $\vec{r}={\left(r_{1},r_{2},.\cdot \cdot \cdot ,r_{n}\right)}^{T}$ (where *n* is the sample length) based on the traditional algorithm. The vector contains the feature information of the objective function proposed, and the information can be changed according to the source signal. This paper uses the mean square to measure the error between reference vectors, namely:

(13) $$\varepsilon (y,\vec{r})=E\{{\left(y-\vec{r}\right)}^{2}\}.$$

When *y* is completely closed to the source signal, $\varepsilon (y,\vec{r})$ has a minimum value. When $\varepsilon (y,\vec{r})$ satisfies Equation (14), the output result *y* is the desired source signal,

(14) $$g(y)=\varepsilon (y,\vec{r})-\xi \leq 0$$

where ξ is the threshold value. Using g(y) as the feasibility constraint of Equation (10), the solution of the algorithm can be projected onto the feasibility constraint function. Therefore, the problem of the improved SNMF algorithm can be summarized as follows:

(15) $$\begin{array}{l} \min\;D(V||WH)=\sum_{ij}[v_{ij}\log\frac{v_{ij}}{{\left(WH\right)}_{ij}}-v_{ij}+(WH{)}_{ij}]+\lambda \sum_{kj}H_{kj}\\ s.t.\;W,H>0,\;\sum_{i}W_{ik}=1,\;\;p(y)=E\{y^{2}\}-1=0 \end{array}$$

where p(y) is the limiting constraint of the objective function, and *y* is the solution vector.

The steps of Algorithm 1 are as follows:

**Algorithm 1:** Improved Sparse Non-Negative Matrix Factorization*Step 1*. Initialize non-negative matrices *W* and *H* randomly*Step 2*. Extract the constraint reference vector $\vec{r}$ with the feature of the source signal*Step 3*. Calculate the initial value of the objective function from Equation (15)*Step 4*. According to Equations (11) and (12), update the matrices ***W*** and ***H*** alternately and iteratively*Step 5*. If the objective function converges, the iteration is stopped, and the matrices ***W*** and ***H*** are outputted; otherwise, steps (3) and (4) are performed cyclically

The biggest advantage of the improved SNMF method is that the vector is added as a constraint reference, which constrains the objective function and can be generated adaptively according to the source signal, and the redundant component after decomposition is reduced.

## 4. Signal Separation Method Based on Improved SNMF

Based on the above analysis, a separation method of compound fault signals with improved sparse non-negative matrix factorization is put forward for the bearings in rotating machinery. The implementation steps Algorithm 2 are summarized as follows:

**Algorithm 2:** Signal Separation Method Based on Improved SNMF*Step 1*. The method of short-time Fourier transform (STFT) is applied to the original vibration signal to obtain a high-dimensional feature matrix that characterizes local information.*Step 2*. Take the energy value of the feature matrix to satisfy the input matrix of improved SNMF.*Step 3*. Use the improved SNMF algorithm to reduce the dimension, and get the base matrix ***W*** and the coefficient matrix ***H***.*Step 4*. The base matrix ***W*** and the coefficient matrix ***H*** are reconstructed in a low-dimensional space, and the time–frequency information is transformed into the time domain by using an inverse time Fourier transform (ISTFT) to obtain a reconstructed waveform of the feature component.*Step 5*. The reconstructed signal is selected for envelope spectrum analysis to extract the fault feature of the bearing.

**Figure 2:**
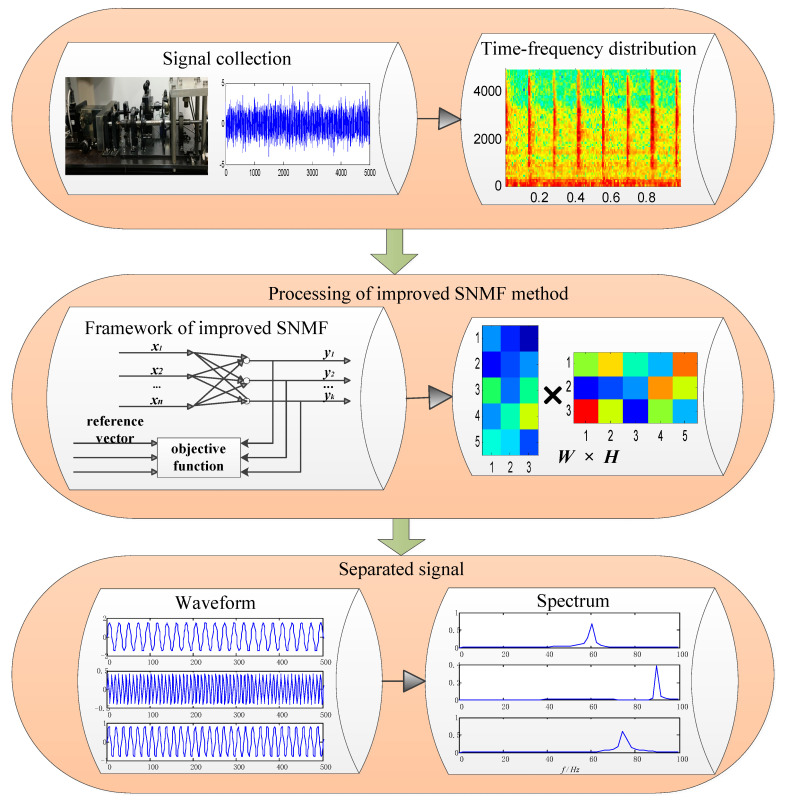
Flowchart of compound faults diagnosis.

The flow chart is shown in Figure 2.

## 5. Verification with Simulation and Experiment

### 5.1. Simulation Analysis

**Figure 3:**
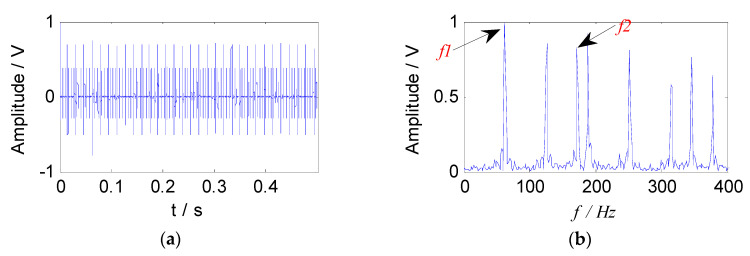
The simulated signal: (**a**) The waveform of signal; (**b**) the envelope spectrum of signal.

In order to verify the effectiveness of the proposed method, the following model was used to simulate compound faults of a rolling bearing:

(16) $$s(t)=e^{-2\pi gf_{n}(t-T)}\sin(2\pi f_{n}\sqrt{1-g^{2}}(t-T))$$

(17) $$S(t)=A{\left[s_{1}(t),s_{2}(t)\right]}^{T}$$

where, *g* = 0.1 is the damping coefficient, $s_{1}(t)$ and $s_{2}(t)$ are composed of the following two feature parameters: The natural frequencies are 3000 Hz and 5000 Hz respectively, the characteristic frequencies are taken as 63 Hz and 173 Hz, the sampling frequency is 100 kHz, and the analysis points are taken as 0.5 s time segments. *A* = [0.8147, 0.9058] is a mixed matrix generated randomly. The mixed signal *S(t)* is obtained by Equation (17). The waveform and the envelope spectrum of the mixed signal are shown in Figure 3.

**Figure 4:**
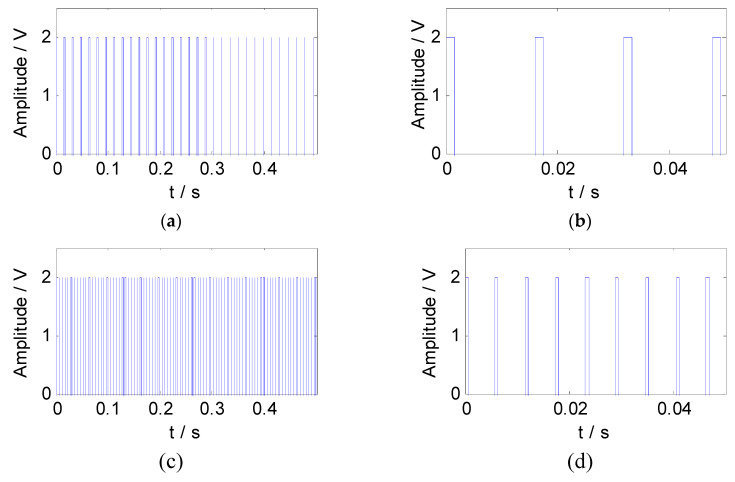
The reference signals of the simulated signal: (**a**) The waveform of reference signal 1; (**b**) the partial enlargement of reference signal 1; (**c**) the waveform of reference signal 2; (**d**) the partial enlargement of reference signal 2.

According to the feature information of the simulated signal, the constraint reference signals were constructed by the pulse method. The square wave sequences with the same length as the mixed signal were generated as the constraint reference signals. The waveform and the partial enlargement of the reference signals are shown in Figure 4.

**Figure 5:**
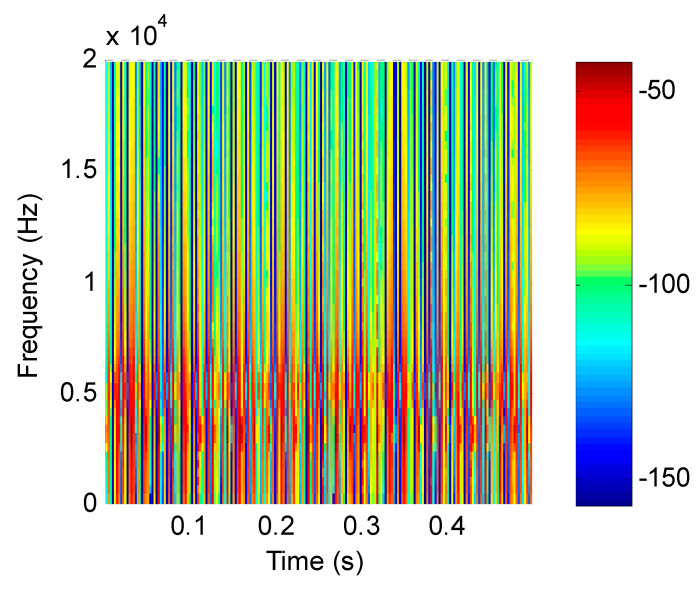
Time–frequency distribution of simulation signals.

For the mixed simulation signal, the proposed method was used for analysis. Firstly, the feature matrix ***X*** was obtained by STFT, and the time–frequency distribution is shown in Figure 5. Secondly, the energy value of the feature matrix was obtained as the input matrix of the improved SNMF. Thirdly, the energy-value matrix was decomposed by the improved SNMF algorithm to obtain the base matrix ***W*** and the coefficient matrix ***H***. Finally, the matrix ***W*** and ***H*** were reconstructed in the subspace, and the inverse short-time Fourier transform was used to transform them into the time domain, getting separated signals.

**Figure 6:**
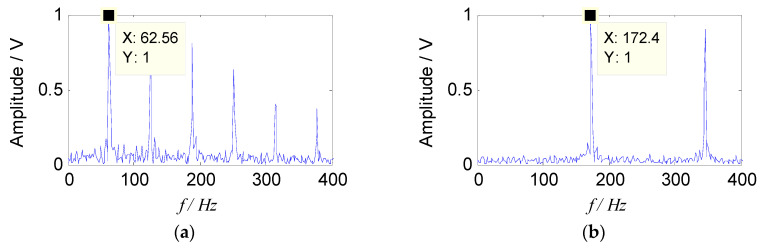
Envelope spectra of separated signals with the improved sparse non-negative matrix factorization (SNMF): (**a**) Envelope spectrum of *s*_1_; (**b**) envelope spectrum of *s*_2_.

By introducing the constraint reference signals, two sets of separated signals were obtained, which indicated that the feature information in the two sets of separated signals was rich, and described the source signal better. The envelope spectra of separated signals are shown in Figure 6.

It can be seen from Figure 6 that the two characteristic components, 63 Hz and 173 Hz, included in the source signal can be separated by the proposed method. Therefore, from the analysis of simulation signal, it can be concluded that the proposed method can effectively separate the source signal from the mixed signals, and the characteristic frequency of the source signal can also be extracted in the envelope spectrum, which verifies the effectiveness of the proposed method.

**Figure 7:**
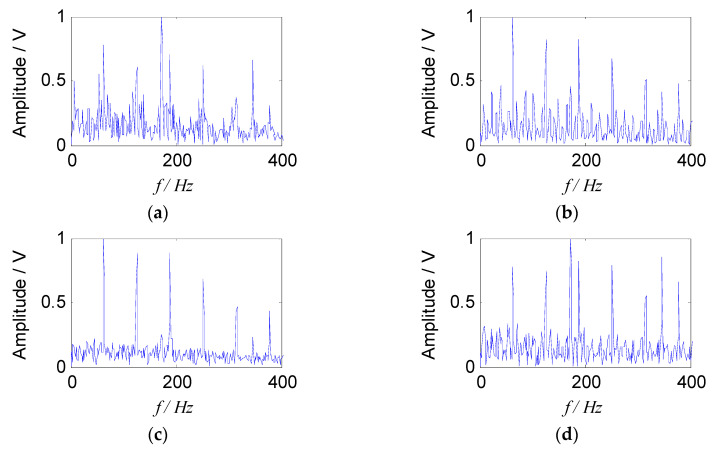
Envelope spectra of separated signals with the traditional SNMF: (**a**) Envelope spectrum of *s*_3_; (**b**) envelope spectrum of *s*_4_; (**c**) envelope spectrum of *s*_5_; (**d**) envelope spectrum of *s*_6_.

In order to verify the advantages of the proposed method, it is compared with the traditional SNMF algorithm using the model of KL-divergence. When the reference vector is not been introduced as a constraint term, some redundant signal components (*s*_3_, *s*_4_, *s*_5_, *s*_6_) are obtained as the Figure 7 shows. It can be seen from the Figure 7 that the spectrogram is disorganized. In order to illustrate quantitatively the advantages of the reference vector, the optimal two sets of signals in the Figure 7 were compared with the separated signals in Figure 6 and used to define the following Equation (18) to quantify the gains of the proposed approach. The SNRs (signal to noise ratio) of two methods are shown in Table 1.

(18) $$SNR=\mathrm{lg}\frac{\sum_{r=1}^{3}A_{F_{r}}{}^{2}}{\sum_{i=1}^{N}A_{i}{}^{2}-\sum_{r=1}^{3}A_{F_{r}}{}^{2}}$$

where *N* is the sampling length, $A_{F_{r}}$ is the amplitude of the first three-order characteristic frequency, and $A_{i}$ is the amplitude of the frequency domain signal.

According to Figure 7 and Table 1, the proposed approach has better separation and noise reduction effects.

### 5.2. Experiment and Discussion

**Figure 8:**
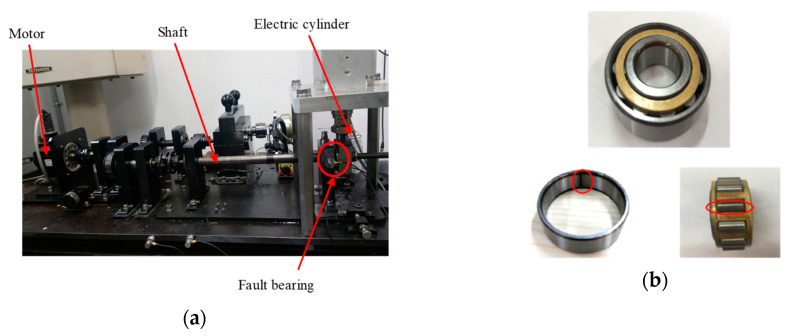
Experimental platform and fault bearing: (**a**) Experimental platform; (**b**) fault bearing.

In this section, the measured compound fault signals of bearing are taken as the research object to verify the effectiveness of the proposed method. The defects of 0.5 mm width and 0.15 mm depth were machined artificially on the outer ring and rolling elements of the bearing. The type of cylindrical roller bearing was NTN N204. During the experiment, the vibration signal in the vertical direction was collected by the acceleration sensor placed on the bearing housing. The experimental platform of the rotating machine and the fault bearing are shown in Figure 8. The sampling frequency was 100 kHz and the sampling time was 10s. The motor speed was set to 900 rpm, and the rolling bearing components were calculated according to the bearing structural parameters (Table 2) and the equations in references [47,48]. The theoretical characteristic frequency is shown in Table 3.

**Figure 9:**
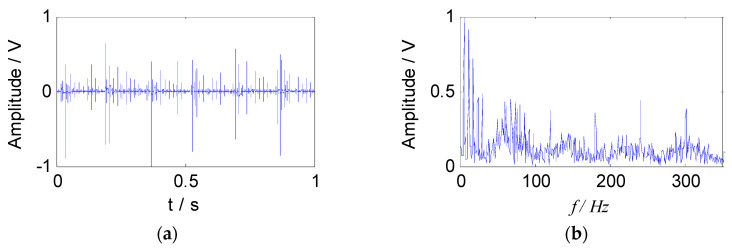
The vibration signal of compound faults at 900 rpm: (**a**) The waveform of signal; (**b**) the envelope spectrum of signal.

The compound fault signals collected were used for analysis, and the analysis points were taken as 1s time segments randomly. The waveform and the envelope spectrum at 900 rpm are shown in Figure 9.

The impact pulse component can be seen clearly from the time domain waveform, indicating that the bearing had failed, but the feature of period time is not obvious, and the useful state information of the bearing could not be obtained. In the envelope spectrum, the defect feature of outer race and rolling element was submerged by the noise component, and it was difficult to identify. The peak appearing at the frequency of about 6 Hz (first peak) in the spectrum, and the frequency value was close to the characteristic frequency of the cage, that is, the revolving frequency of the rolling element, so the peak was caused by the impact of the rolling elements.

**Figure 10:**
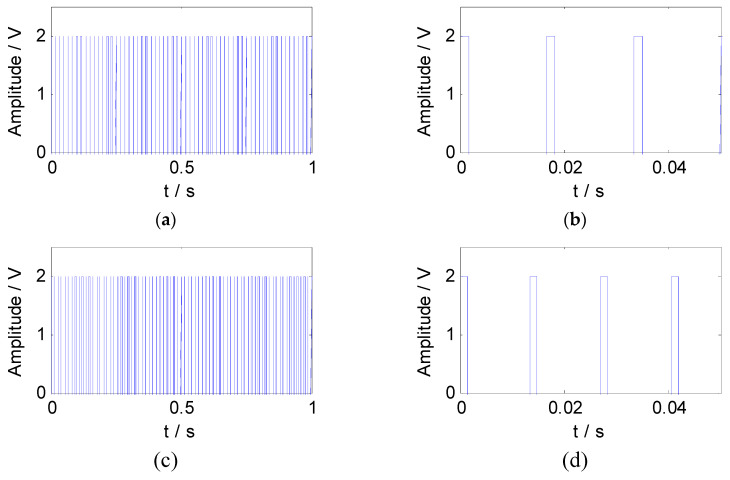
The reference signals of experimental signal: (**a**) The waveform of reference signal 3; (**b**) the partial enlargement of reference signal 3; (**c**) the waveform of reference signal 4; (**d**) the partial enlargement of reference signal 4.

According to the feature information of the experimental signal, the constraint reference signals were constructed by the pulse method. The square wave sequences with the same length as the experimental signal were generated as the constraint reference signals. The waveform and the partial enlargement of the reference signals are shown in Figure 10.

**Figure 11:**
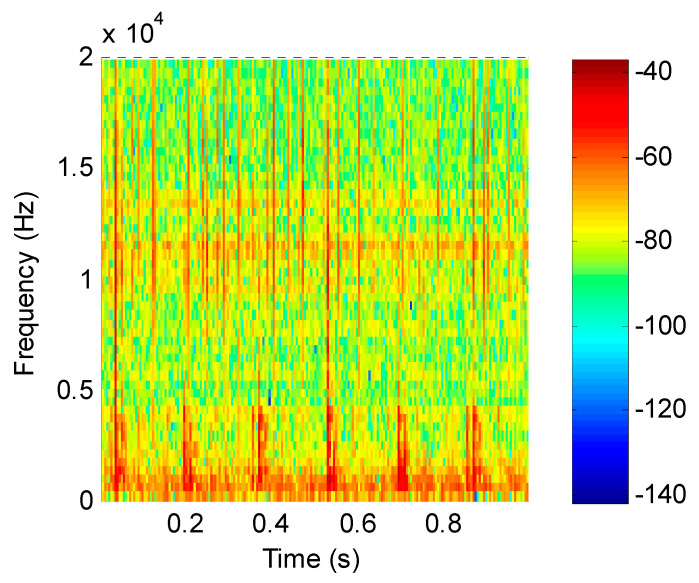
Time–frequency distribution of the collected signal.

According to the proposed method, the original signal was subjected to short-time Fourier transform to obtain a feature matrix ***X***, and the time–frequency distribution is shown in Figure 11. The energy value of the feature matrix was obtained as an input matrix of the improved SNMF, then the energy-value matrix was decomposed by the improved SNMF algorithm to obtain the base matrix ***W*** and the coefficient matrix ***H***. Finally, the matrix ***W*** and ***H*** were reconstructed in the subspace, and the inverse short-time Fourier transform was used to transform them into the time domain, getting reconstructed signals.

**Figure 12:**
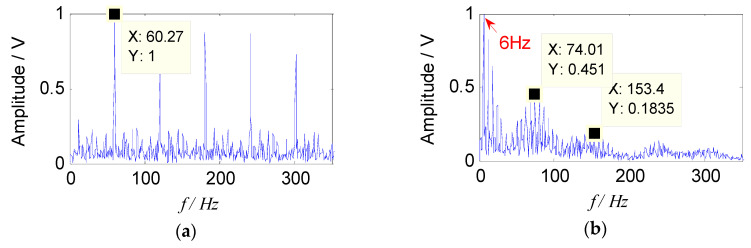
Envelope spectra of separated signals with the proposed method: (**a**) Envelope spectrum of outer-race fault; (**b**) envelope spectrum of roller fault.

Two sets of separated signals could be obtained by introducing a constrained reference vector, which indicated that the feature information in the two sets of separated signals was rich, and the source signal contained two fault components. The envelope spectra of separated signals are shown in Figure 12.

It is obvious that two source signal components were obtained by the proposed method, which corresponded to a fault characteristic frequency of the outer race and the rolling element respectively, and they were consistent with the theoretical characteristic values. Meanwhile, their higher harmonic components were also extracted clearly. In addition, the cage frequency (6 Hz) and its higher harmonic components appear in the Figure 12b, and the sidebands of the fault characteristic frequency were distributed by the cage frequency, which was consistent with the feature of the rollers failure. Therefore, the results show that the proposed method could effectively separate the fault source signal from the mixed signals, and the fault characteristic frequency could also be extracted in the envelope spectrum, which verified the effectiveness of the proposed method in the field of compound fault diagnosis of bearings.

### 5.3. Comparison with Traditional Method

**Figure 13:**
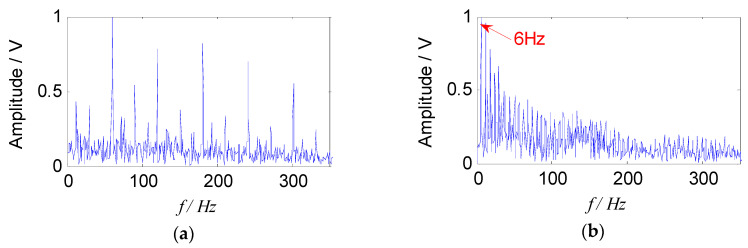
Envelope spectra of separated signals with the SNMF method: (**a**) Envelope spectrum of *f*_1_; (**b**) envelope spectrums of *f*_2_.

In order to verify the advantages of the proposed method in the field of compound faults diagnosis of bearings, it was compared with the traditional SNMF algorithm using the model of KL-divergence. The experimental data at 900 rpm was selected to illustrate this. The energy value of the feature matrix obtained by the short-time Fourier transform, and the traditional SNMF algorithm was used to reduce the dimension. The matrix ***W*** and ***H*** were reconstructed in the subspace, selecting the reconstructed signal as the separated signal (*f*_1_ and *f*_2_), and the envelope spectra are shown in Figure 13.

It can be seen from Figure 13 that the compound fault signals were not separated effectively using the traditional SNMF algorithm based on KL-divergence. The fault feature of outer race was almost extracted, and the fault feature of rolling element was submerged. In addition, the cage characteristic frequency (6 Hz) could only be obtained in the Figure 13b, failing to describe the fault source signal accurately. The proposed method can extract and separate the fault features of the rollers and the outer race effectively. Comparing Figure 13 and Figure 12, it can be seen that since the improved SNMF algorithm enhances the local features and sparsity of the fault component, and reduce the redundant information of the reconstructed signal, the source signal can be separated, and the feature can be extracted. The unique advantages of the proposed method in the field of compound fault diagnosis of bearings have been proved.

## 6. Conclusions

The feature of compound fault signals in rotating machinery is weak, and the source signals of the compound fault are difficult to separate from the mixture signal. Aiming to solve these problems, a blind source separation method with single channel based on the improved SNMF was proposed. The square wave sequences with the feature information were constructed as the constraint reference vector into the objective function of the traditional SNMF algorithm, to reduce the redundancy of the decomposed data. STFT was applied to obtain a high-dimensional feature matrix. Considering the high dimensional feature matrix after the STFT, the improved SNMF is adopted to select local feature from time–frequency distribution, and it can lower the dimension of the problem. Meanwhile, according to the local learning ability of the improved SNMF algorithm, the compound fault features can be separated effectively, and the redundant component after decomposition is reduced on the basis of effective data reduction. The simulation and test results show that the proposed method can effectively separate the feature of compound faults for roller bearing. Compared with the traditional SNMF, the feature extraction and separation capability of the proposed method is superior to the traditional SNMF. Therefore, the proposed method is of great significance for the compound faults diagnosis of rotating machinery and has certain engineering application value. Considering the impact of initialization about the improved SNMF, this paper only initialized randomly to test the performance of the algorithm. The optimized initialization will be studied in the future.

## Figures and Tables

**Table 1:** SNRs of two methods (dB).

The improved SNMF	−0.2527	−0.3421
The traditional SNMF	−0.8484 (Figure 7c)	−1.6449 (Figure 7d)

**Table 2:** Structure parameters of NTN N204 bearing.

Bearing Type	NTN N204
Inner Diameter	20 mm
External Diameter	47 mm
Roller Diameter	6.5 mm
Width	14 mm
Number of Rollers	10
Contact angle	0 rad

**Table 3:** Fault characteristic frequencies.

Fault types	outer race	roller	cage
Characteristic frequencies	60 Hz	74 Hz	6 Hz
